# Force Transmission Modes of Non-Cohesive and Cohesive Materials at the Critical State

**DOI:** 10.3390/ma10091014

**Published:** 2017-08-31

**Authors:** Ji-Peng Wang

**Affiliations:** Building Architecture and Town Planning Department (BATir), Université Libre de Bruxelles, Avenue F.D. Roosevelt 50, CP 194/2, 1050 Brussels, Belgium; Ji-Peng.Wang@ulb.ac.be or Ji-Peng.Wang@outlook.com

**Keywords:** granular material, discrete element modeling, contact model, capillary effect, critical state, force transmission

## Abstract

This paper investigates the force transmission modes, mainly described by probability density distributions, in non-cohesive dry and cohesive wet granular materials by discrete element modeling. The critical state force transmission patterns are focused on with the contact model effect being analyzed. By shearing relatively dense and loose dry specimens to the critical state in the conventional triaxial loading path, it is observed that there is a unique critical state force transmission mode. There is a universe critical state force distribution pattern for both the normal contact forces and tangential contact forces. Furthermore, it is found that using either the linear Hooke or the non-linear Hertz model does not affect the universe force transmission mode, and it is only related to the grain size distribution. Wet granular materials are also simulated by incorporating a water bridge model. Dense and loose wet granular materials are tested, and the critical state behavior for the wet material is also observed. The critical state strength and void ratio of wet granular materials are higher than those of a non-cohesive material. The critical state inter-particle distribution is altered from that of a non-cohesive material with higher probability in relatively weak forces. Grains in non-cohesive materials are under compressive stresses, and their principal directions are mainly in the axial loading direction. However, for cohesive wet granular materials, some particles are in tension, and the tensile stresses are in the horizontal direction on which the confinement is applied. The additional confinement by the tensile stress explains the macro strength and dilatancy increase in wet samples.

## 1. Introduction

Granular materials, composed of solid particles, are common materials in industry and nature, such as powders, glass beads and sands. Microscopically, the mechanical behavior of a granular material can be characterized by its force transmission through inter-particle contacts. Force chains in granular assemblies were firstly observed in the laboratory by photoelastic experiments [1]. The carbon paper technique was also employed to measure forces on boundaries [2]. The probability density distribution of forces on particles was analyzed by researchers, and it was found that there is a certain function for this distribution at the static state [2,3,4].

With the development of the discrete element method [5], a discontinuous method can simulate individual particles, and more detailed inter-particle interactions can be investigated both at the initial static state and during material deformation (for example, in the triaxial test). It has been observed that the probability density distribution of normal contact forces is generally in an exponential law [6,7]. With the increase of deformation, the distribution of contact forces evolves correspondingly. The probability of weak forces (forces less than the average) is increased with material deviatoric stress. When the material is sheared to a large deformation, the force distribution pattern also becomes constant [8,9]. This could be related to the critical state [10], which is an important definition for granular material mechanics. When a material is sheared to a large deformation, the material strength and void ratio tend to be constant independent of its initial relative density. The unique critical state behavior implies that the internal structure and force transmission pattern could also follow a certain rule, and this should be explored to understand the micro-macro relationship. However, the previous study is mainly based on one kind of material without comparing with the result of a material with a different void ratio ($e=\frac{\Phi }{1-\Phi }$, where Φ is the solid fraction). To discover the critical state force transmission mode, it is essential to simulate materials with different initial relative densities and also different particle size distributions.

As the DEM simulation is a numerical approach, the internal force transmission and thus the macro behavior of a simulated granular material may be highly dependent on the inter-particle contact law. Typically, the linear Hooke contact law and the non-linear Hertz contact model are two widely-used contact laws for non-cohesive granular materials. To study the critical state force transmission pattern, a comparison between the two contact models cannot be neglected. Moreover, attractive contact models may also be applied in DEM to simulate cohesive granular materials; such as the simple linear cohesive models [11] and capillary bridge models [12,13,14,15,16], in which the effect of water bridges between particles is represented. With the capillary water bridge effect, the force transmission pattern of wet granular materials also attracted research interest in the past decade [17,18]. It has been observed that the cohesive effect between particles alters the static state force distribution obviously, and the tensile force between grains plays a vital role in material strength. For wet granular materials, the critical state also exists and is also a very important characteristic for the material constitutive laws [19,20,21]. Therefore, the microscopic behaviors of cohesive granular materials at the critical state, including force and stress transmission modes, should also be investigated.

In this paper, non-cohesive dry granular materials and cohesive wet granular materials are simulated by DEM, and the contact model effect on the critical state force transmission mode is analyzed. For dry granular material, both the linear Hooke contact model and the non-linear Hertz contact model are applied. Relatively loose and dense materials with two different grain size distributions are sheared from an isotropic state to the critical state. The force transmission pattern at critical state is then analyzed mainly by its probability density distribution. For wet granular materials, a water bridge model is employed to simulate the cohesive effect, and the force and stress transmission modes of wet granular materials are compared with those of dry materials following the same loading path. These may confirm the unique critical state force transmission modes for dry and wet granular materials, respectively, and the contact model effect can also be revealed with a better understanding of the link between micro interactions and macro behaviors.

## 2. Discrete Element Modeling

### 2.1. Brief Introduction of the Discrete Element Method

The DEM method is an explicit time stepping algorithm by applying the basic Newton’s second law of motion. In the DEM simulation, particles are assumed as rigid bodies, and inter-particle deformations are very small and thus can be simplified as inter-particle overlaps. The time step is tiny enough that the velocities and accelerations of particles can be simplified as constant values during one time step. Then, the interactive forces and moments between particles are calculated by contact laws, on the basis of inter-particle overlaps and parameters associated with particle material properties. For the simulation of cohesive materials, such as wet granular materials, attractive forces may be added between particles to modify the existing contact laws by following certain physical criteria.

Rotations and velocities of particles obey Newton’s second law. The simulation starts from an assembly of particles with known grain material properties, geometries, orientations, initial positions and velocities. By inputting the material properties (i.e., particle stiffness and friction coefficient), inter-particle velocities and the inter-particle overlaps to the contact law, the inter-particle forces and moments can be solved. Then, the resultant inter-particle forces and moments are substituted into Newton’s second law to obtain the updated velocities and positions by the time step integration. Cycle by cycle, the deformation (i.e., a triaxial shearing) of a granular material assembly can be simulated. This gives a good connection between the microscopic forces and the macroscopic granular material behaviors for geomechanics research. In this study, the open source DEM software LIGGGHTS [22] is employed. LIGGGHTS stands for LAMMPS improved for general granular and granular heat transfer simulations. LAMMPS is a classical molecular dynamics simulator. LIGGGHTS improves the granular package leading the qualified granular material simulation performances. Since the source code is open, it makes it easier to add a new capillary bridge model.

### 2.2. Linear Hooke’s Contact

The inter-particle contact law plays a vital role in DEM simulations, as it governs the inter-particle forces and thus affects the material behaviors. For cohesionless granular materials, there are two main contact laws widely applied, which are the linear Hooke contact model and the non-linear Hertz contact model. These two basic contact laws are included in the LIGGGHTS software, which can be employed to simulate dry granular materials. In Hooke’s model, the inter-particle contact is simplified as a spring-dashpot system in which the spring represents the elastic contact force, and the dashpot governs the damping effect. For two particles in contact, the elastic force is in a linear relationship with the inter-particle deformation, and the damping force is determined by the damping ratio and the relative velocity.

**Figure 1:**
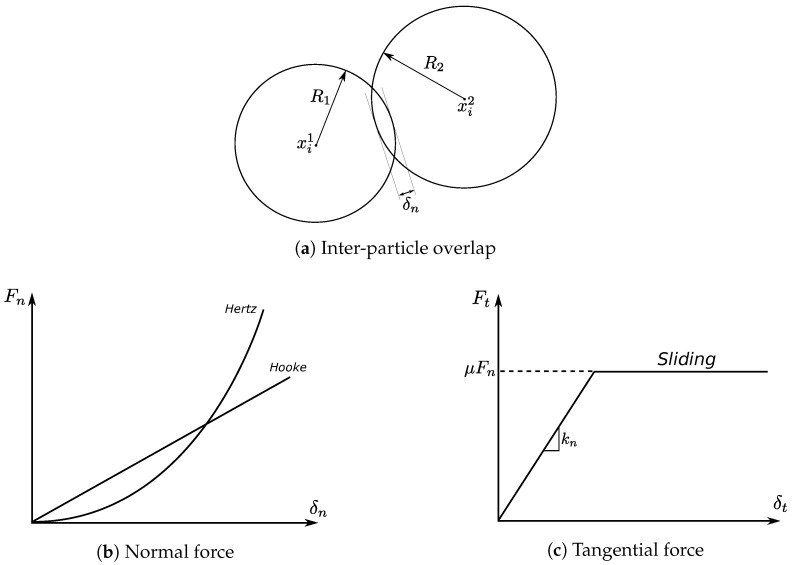
Overlap and inter-particle forces in the Hooke and Hertz contact laws.

In DEM, the inter-particle deformation is represented by the inter-particle overlap, as the spherical balls are rigid. It is defined as in Figure 1a that $\delta_{n}=R_{1}+R_{2}-\left|x_{i}^{1}-x_{i}^{2}\right|$. The total normal inter-particle force (its absolute value) is formulated as:

(1) $$F_{n}=-k_{n}\delta_{n}+\gamma_{n}\Delta v_{n}$$

where $k_{n}$ is the normal contact stiffness between the two particles, $\delta_{n}$ is the normal inter-particle deformation, $\gamma_{n}$ is the normal damping coefficient and $\Delta v_{n}$ represents the normal inter-particle relative velocity. In a quasi-static deformation, i.e., the strain rate is relatively low, the damping force is much smaller than the elastic force. Thus, the normal contact force is normally in a linear relationship with the inter-particle overlap (Figure 1b).

The tangential force (its absolute value) is also based on a spring-dashpot system for which the elastic tangential force is related to the tangential relative displacement, and the damping force is related to the tangential relative velocity:

(2) $$F_{t}=\int_{t_{0}}^{t}k_{t}\Delta v_{t}dt+\gamma_{t}\Delta v_{t}$$

where $k_{t}$ is the tangential contact stiffness, $\gamma_{t}$ is the tangential damping coefficient and $\Delta v_{t}$ represents the tangential inter-particle relative velocity. The integration $\int_{t_{c,0}}^{t}\Delta v_{t}dt$ represents the relative displacement in the tangential direction from the time when the two particles in contact $t_{0}$ to the current time *t*. In DEM simulations, the above equation represents a tangential inter-particle force in a static state. When inter-particle sliding occurs, the tangential force also follows the Coulomb friction law, so that:

(3) $$F_{t}\leq \mu F_{n}$$

in which μ is the inter-particle friction coefficient. The evolution of the tangential force against the tangential displacement $\delta_{t}$ is illustrated in Figure 1c.

### 2.3. Non-Linear Hertz Contact

In the Hertz model, the inter-particle force formulation is based on solid contact mechanics by using particle material properties, including Young’s modulus *E*, Poisson’s ratio ν and the coefficient of restitution $e_{r}$, as input parameters. Following [23,24], the normal elastic force for two spherical particles in contact can be expressed as:

(4) $$F_{n}=\frac{2\sqrt{2}}{3}R^{2}E^{*}{\left(\frac{\delta_{n}}{R}\right)}^{3/2}$$

where $E^{*}$ is the effective Young’s modulus and *R* is the particle radius. In the case that the particles are not the same size, the harmonic mean is applied to *R* as $R=\frac{2R_{1}R_{2}}{R_{1}+R_{2}}$. The effective Young’s modulus for the two particles in contact is calculated by:

(5) $$\frac{1}{E^{*}}=\frac{1-\nu_{1}^{2}}{E_{1}}+\frac{1-\nu_{2}^{2}}{E_{2}}$$

where $E_{1}$, $E_{2}$ and $\nu_{1}$, $\nu_{2}$ are the Young’s moduli and Poisson’s ratios for Particle 1 and Particle 2; such that the normal force has a non-linear relation with the inter-particle deformation (Figure 1b).

In LIGGGHTS, when the non-linear Hertz model is applied, the contact stiffness $k_{n}$ in Equation (1) is no longer a constant, but is related to the inter-particle deformation (inter-particle overlap). When using the Hertz model, $k_{n}$ is replaced by:

(6) $$k_{n}=\frac{2\sqrt{2}}{3}E^{*}\sqrt{R\delta_{n}}$$

Based on the inter-particle collision study by [25,26,27], the damping force is associated with the coefficient of restitution of the particle material. In the LIGGGHTS implementation, when the Hertz model is used, the normal damping coefficient in Equation (1) is determined by relevant material parameters as:

(7) $$\gamma_{n}=-\sqrt{\frac{10}{3}}\frac{ln(e_{r})}{\sqrt{ln^{2}\left(e_{r}\right)+\pi^{2}}}\sqrt{E^{*}m}{\left(\frac{R\delta_{n}}{2}\right)}^{1/4}$$

where $e_{r}$ is the coefficient of restitution and *m* is the particle mass. When the masses of the two particles are different, a harmonic mean is applied as $m=\frac{2m_{1}m_{2}}{m_{1}+m_{2}}$ (the contact forces are calculated by the harmonic mean radius). This is to reduce the calculation time for grains with unequal sizes. Verifications of the harmonic mean can be seen in [28,29], which show that the error is very small.

By [30], the elastic tangential contact force for the tangential motion before sliding is derived as:

(8) $$F_{t}=\int_{t_{0}}^{t}8G^{*}\sqrt{\frac{R\delta_{n}}{2}}\Delta v_{t}dt$$

where $\Delta v_{t}$ is the tangential inter-particle relative velocity and $G^{*}$ is the effective shear modulus between Particle 1 and Particle 2, which is defined as:

(9) $$\frac{1}{G^{*}}=\frac{2\left(2+\nu_{1}\right)\left(1-\nu_{1}\right)}{E_{1}}+\frac{2\left(2+\nu_{2}\right)\left(1-\nu_{2}\right)}{E_{2}}$$

Thus, when the Hertz model is applied, the tangential contact stiffness in Equation (2) is determined by the material property and the particle deformation as:

(10) $$k_{t}=8G^{*}\sqrt{\frac{R\delta_{n}}{2}}$$

Similar to the normal damping coefficient, the tangential damping coefficient $\gamma_{t}$ is also determined by the coefficient of restitution and other relevant material parameters as:

(11) $$\gamma_{t}=-2\sqrt{\frac{10}{3}}\frac{ln(e_{r})}{\sqrt{ln^{2}\left(e_{r}\right)+\pi^{2}}}\sqrt{G^{*}m}{\left(\frac{R\delta_{n}}{2}\right)}^{1/4}$$

### 2.4. Capillary Bridge Model

**Figure 2:**
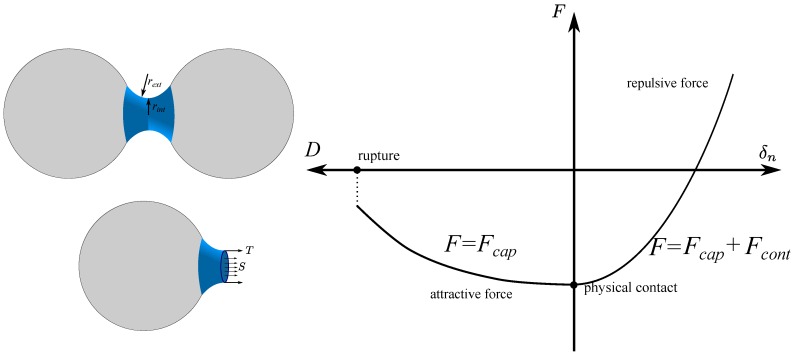
Capillary bridge model.

A capillary bridge model can be embedded in DEM to simulate wet granular materials. The capillary bridge model in [31] is adopted in this research (Figure 2). It should be noted that this model is only valid for materials with a relatively low degree of saturation (the pendular state) as with higher water content, water bridges may coalesce with each other [32]. In this capillary bridge model, it is assumed that pendular water bridges are formed between neighboring particles. The matric suction (denoted as *S*), which is the pressure difference between the air and water phase ($u_{a}-u_{w}$), is maintained constant throughout the sample. The water bridges are in toroidal shapes, which means the meridian profile of a water bridge surface is simplified as a circular arc. By the Young-Laplace equation, the matric suction has a relationship with the capillary bridge curvature and the water surface tension as:

(12) $$S=T\left(\frac{1}{r_{ext}}-\frac{1}{r_{int}}\right)$$

where *T* is the water surface tension as a physical constant (T=0.073 N/m for distilled water), $r_{ext}$ is the external radius of the toroidal shape meridian profile and $r_{int}$ is the internal radius of the water bridge at the neck. The capillary force is calculated by the `gorge method’ [33], as the sum of the pressure difference acting on the section of the bridge neck and the surface tension acting on the water-air interface:

(13) $$F_{cap}=S\pi {r_{int}}^{2}+T\left(2\pi r_{int}\right)$$

In this capillary model, a water bridge may exist between two neighboring particles with a small gap, *D*. By knowing the particle geometry, matric suction, inter-particle distance and the water-air-solid contact angle (idealized as zero in this study), the geometry of the water bridge ($r_{int}$ and $r_{ext}$) can be solved iteratively [31]. Thus, the capillary force is obtained from the above equation. The water volume of the capillary bridge can be calculated by an integration of the water bridge profile, based on which the degree of saturation of an assembly can be calculated.

The capillary bridge model consists of three conditions. When the inter-particle distance is too large (beyond the rupture distance), the numerical solution does not exist, and there is no inter-particle interaction. When the inter-particle distance is within the rupture distance, and the two particles are not in physical contact, the inter-particle force is the capillary force. Additionally, if the particles are in physical contact, the inter-particle normal force is the sum of the attractive capillary force and the repulsive mechanical contact force. In this study, the non-linear Hertz law is employed to model the physical contacts in the investigation of wet granular materials.

### 2.5. Model Parameters and Sample Preparation

**Figure 3:**
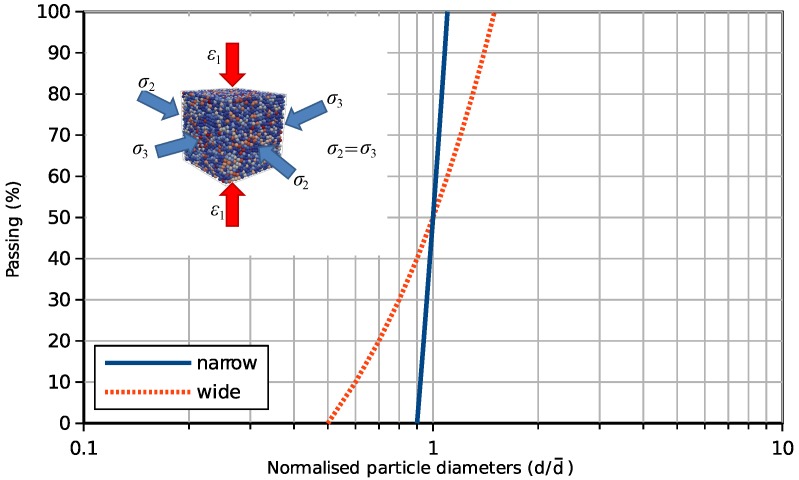
Particle size distributions.

By using the contact laws introduced above, dry and wet granular specimens will be simulated in a cubic representative volume element (RVE) surrounded by smooth boundaries with the edge length 20-times the mean particle diameter. This leads the total particle number in one specimen to be around 9000 dependent on the relative density and particle size distribution. In this study, the mean particle diameter is 0.02 mm. There are two types of particle size distributions (PSDs) in this research, a narrow type and a wide type. Both of them are uniform distributions in weight. The narrow PSD ranges from 0.9–1.1-times the mean particle diameter and the wide type ranges from 0.5–1.5-times the mean size (Figure 3). The inter-particle friction coefficient μ is set as 0.5. The density of the particles is 2500 kg/m${}^{3}$.

In the study of the critical state force transmission modes of dry granular materials, both the narrow and wide PSDs are investigated. For the wet granular material investigation, the narrow PSD is focused on. Model parameters related to the mechanical contact models (Hertz and Hooke) are summarized in Table 1. For the Hertz contact, the Young’s modulus of the particles is set as 70 GPa, and the Poisson’s ratio ν is 0.25, according to the property of quartz. The restitution coefficient $e_{r}$, which is a physical parameter related to energy dissipation during the inter-particle collision, is set to 0.2. The restitution coefficient *e* is usually defined as the ratio between the relative speed after the collision and the relative speed before the collision. Additionally, for the Hooke contact model, the normal and tangential contact stiffness is $10^{5}$ N/m, and the damping ratio is set to be 0.7.

The cubic samples are prepared by using the typical radius expansion method [34,35]. Particles are firstly generated with a reduced geometry without physical contacts, and then, the particles are expanded to the target PSD. To prepare the relatively dense and loose samples, different inter-particle friction coefficients are used instead of 0.5 during the expansion process. After radius expansion, the friction coefficient is reset to 0.5 for all specimens. Different initial inserted solid fraction values are tried to make sure that after the radius expansion process, the stress on the boundary is just about to increase (this is corresponding to the jamming state [36,37]). This is to avoid any pre-consolidation on the samples. In this study, the contact model effect on the force transmission mode will be studied. This requires the same initial void ratio for both the Hooke model sample and the Hertz model sample. For the narrow PSD, the dense samples for the two contact models are prepared by setting the inter-particle friction coefficient as zero. The void ratio for the dense samples is about 0.63 after the sample preparation. To prepare loose samples with similar void ratios, during the radius expansion process, the inter-particle friction coefficient for the Hooke model is set as 0.3, and the friction coefficient for the Hertz model is 0.9. Then, after the preparation, the two samples reached similar void ratios around 0.73. The samples with the wide PSD are prepared in the same way, and after radius expansion, the void ratios for the dense and loose samples are 0.54 and 0.63, respectively.

After sample preparation, the cubic samples are compressed to a certain mean stress isotropically. Triaxial tests are then carried out by following the conventional triaxial loading path. The lateral stress is maintained constant while deformation is applied in the axial direction. A servo-control technique is used to maintain the stress by adjusting the boundary displacement to reach the equilibrium state of the desired stress. In order to avoid the dynamic effect, the quasi-static equilibrium should be maintained in each time step. The quasi-static condition is judged by the unbalance force ratio, defined by the ratio between the average unbalance force of the particles and the average inter-particle force. The unbalance force ratio is maintained to be less than 0.01 during triaxial loading by adjusting the axial strain rate. When it is over 0.01, the axial loading is stopped for a certain amount of time steps until the forces on particles are rebalanced.

## 3. Force Transmission of Dry Granular Materials

### 3.1. Triaxial Test from the Same Dimensionless Stress State

According to [28,38,39], by using dimensionless input parameters, under quasi-static conditions, the DEM simulation results are the same despite the scale. This means for the same contact model, if two samples are geometrically similar, by using the dimensionless input parameters, the mechanical response (presented in dimensionless numbers) in the triaxial test should be the same. To compare the contact model effect, it is better to use dimensionless parameters to avoid any unit and scale effect. Confining stress is the only input parameter with a dimension (the pressure unit), samples modeled by different contact laws should have the same dimensionless stress before triaxial shearing.

For the Hooke contact model, the dimensionless stress can be expressed by the mean particle contact stiffness $\bar{k_{n}}$ (N/m) and the mean particle radius $\bar{R}$ as:

(14) $$\sigma_{ij}^{*}=\frac{\sigma_{ij}\bar{R}}{\bar{k_{n}}}$$

For the Hertz contact model, the relationship between the inter-particle overlap and contact force is non-linear. By substituting Equation (6) into Equation (14), the dimensionless form stress can be expressed as:

(15) $$\sigma_{ij}^{*}=\frac{\sigma_{ij}\bar{R}}{\frac{4}{3}E^{*}\sqrt{\frac{1}{2}\bar{R}\bar{\delta_{n}}}}$$

where $\bar{\delta_{n}}$ is the mean inter-particle overlap in the assembly.

**Figure 4:**
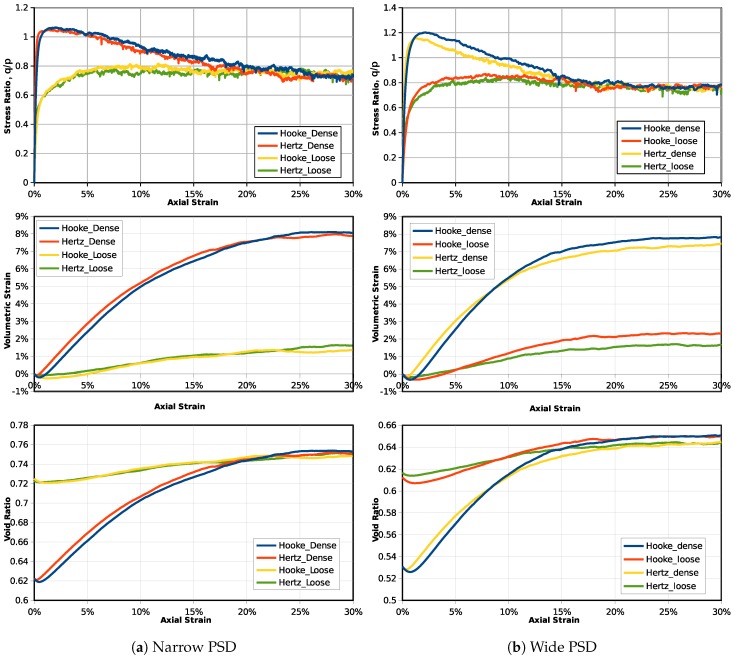
Stress-strain behaviors for samples sheared from the same dimensionless state.

The samples simulated by Hooke and Hertz models are firstly compressed to the same dimensionless stress as $\sigma_{1}^{*}=\sigma_{2}^{*}=\sigma_{3}^{*}=0.001$. Then, the confining stress ($\sigma_{2}^{*}$ and $\sigma_{3}^{*}$) is maintained, and the axial deformation is applied. Figure 4 illustrates the stress-strain behaviors of samples simulated by the two contact models, including dense and loose specimens for both the narrow and wide particle size distributions. The stress ratio (ratio between deviatoric stress and mean stress as q/p where $q=\sigma_{1}-\sigma_{3}$ and $p=(\sigma_{1}+\sigma_{2}+\sigma_{3})/3$) indicates the material strength. It can be seen that the dense specimens have a higher peak strength. However, at large deformation (30%), the ultimate stress ratio values converge to the same, which reflects the existence of the critical state. As the samples are sheared from the same dimensionless stress state, it can be seen that using the Hooke or Hertz contact model for dry granular materials does not affect the stress ratio obviously. For each particle size distribution, there is a critical state stress ratio value independent of the initial void ratio. For the volumetric strain behaviors, it can be observed that the dense specimens are more dilative as they have a higher ultimate volume. The contact model effect on volumetric strain is not obvious in the narrow PSD. However, for the wide PSD, it can be seen that the volumetric strain for the Hooke model samples is slightly higher. Similarly, in terms of the void ratio, there is a unique critical state void ratio for the narrow PSD, but for the wide PSD, the Hooke model samples have a higher ultimate (critical state) void ratio. For the Hooke contact model, the contact stiffness is the same value for all particles. However, based on the physical contact, as Equation (6), the particle contact stiffness is related to its particle radius. For the narrow PSD, particles have similar sizes, which can reduce the discrepancy between the two models, but for a granular material with higher polydispersity, the difference can be larger.

### 3.2. Probability Density Distribution of Overlaps and Forces

**Figure 5:**
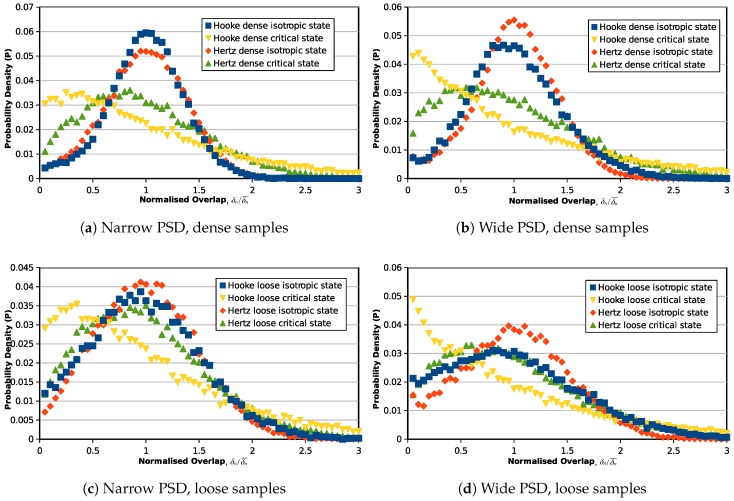
Probability density distribution of normalized overlaps ($\delta_{n}/\bar{\delta_{n}}$).

**Figure 6:**
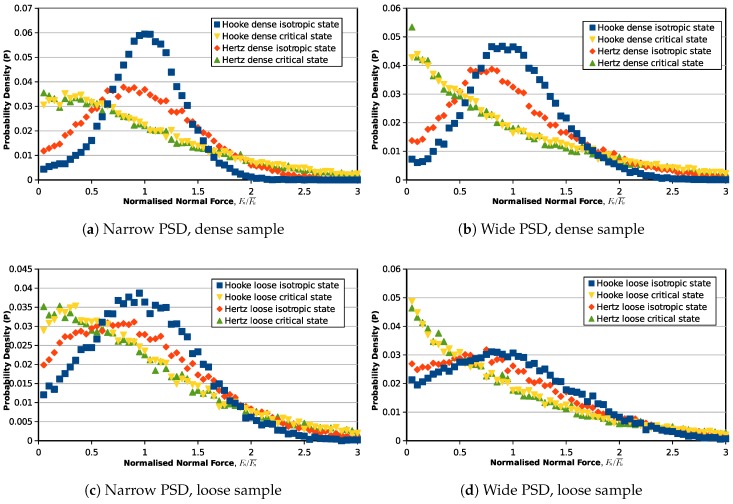
Probability density distribution of normalized normal contact forces ($F_{n}/\bar{F_{n}}$).

**Figure 7:**
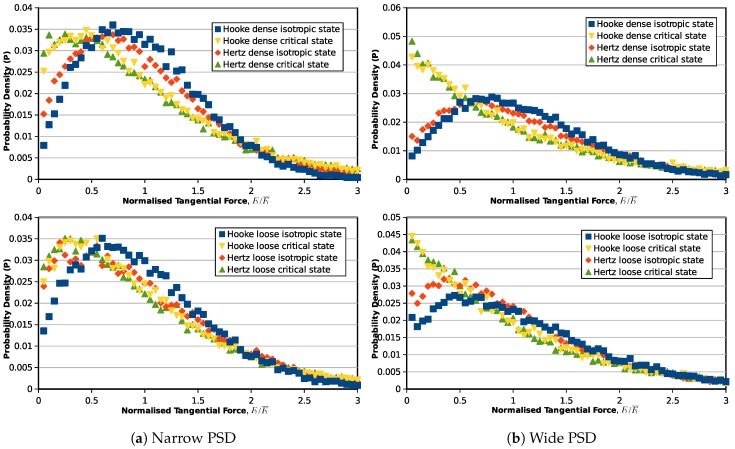
Probability density distribution of normalized tangential contact forces ($F_{t}/\bar{F_{t}}$).

Furthermore, statistical studies are also carried out on the inter-particle overlaps, normal and tangential forces to investigate the force transmission modes from the initial state (0% axial strain) to the critical state (30% axial strain). Figure 5 illustrates the probability distribution of the inter-particle overlapping distance for the samples with narrow and wide PSDs: the horizontal axis represents the overlap normalized by the average overlap value in the assembly, and the vertical axis is the probability of the particular overlap value in the granular assembly. The same probability distribution figure is also plotted for the normalized inter-particle normal forces and tangential forces in Figure 6 and Figure 7. The probabilities of the symbols are calculated on 60 intervals from 0–3.

For dense samples (narrow and wide PSDs) at the isotropic state (initial state), both the Hooke and Hertz models give a normal-distribution-like overlap probability density distribution, with the peak occurring at the average overlap value. The distributions are close to each other for the narrow PSD samples, but the differences become larger when the particle size gradation spreads wider. For loose samples, the contact overlap probability distributions at the initial state are wider than those of dense samples. When the assemblies are sheared to the critical state, the overlaps will be converted to a distribution with more smaller overlaps. Whether a narrow or wide PSD, the critical state overlap distribution is related to the contact model. The Hertz model has a lower probability for small normalized overlaps.

Due to the contact model difference, a similar distribution in contact overlaps will inevitably yield different distributions of contact forces. In Figure 6, the isotropic state normal force distributions for the Hooke contact model samples are similar to their inter-particle overlap distributions because normal contact forces and overlaps are in a linear relationship. For the Hertz model, the distribution is flatter, and there will be a higher amount of weak normal forces at the isotropic state. Although, at the isotropic state, the normal forces have different distribution patterns in different contact models, at the critical state, the contact force distributions for the samples with different contact models and initial void ratios are translated to a similar curve. This phenomenon is valid in both the narrow and wide PSDs, but samples with a wider PSD have more weak forces when it reaches the critical state. Similarly, the tangential contact forces of samples modeled by Hooke and Hertz contact laws start from different probability density distributions at the initial state, but end with the same critical state distribution after the triaxial shearing.

### 3.3. The Unique Force Transmission Pattern at the Critical State

**Figure 8:**
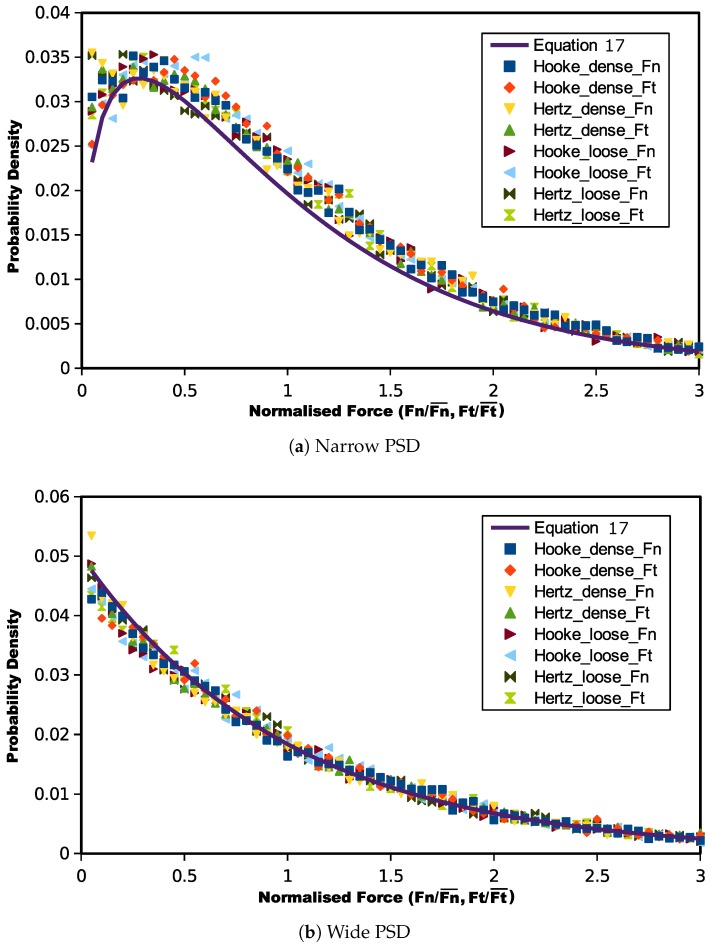
Unique critical state contact force distribution of different contact models.

By comparing Figure 6 and Figure 7, although the initial normal and tangential inter-particle force distribution patterns are not consistent, their critical state probability distributions are analogous to each other. Figure 8 illustrates the normal and tangential contact force distributions together for the two particle size distributions. It can be observed that at critical state, there may be one unique kind of contact force distribution for both normalized normal and tangential forces, independent of the initial void ratio and contact model. The only factor controlling the critical state contact force probability function is the particle size gradation.

The main trend of the critical state contact force distribution for the narrow PSD samples is to ascend, indicating that the stress is mainly transmitted by weak forces. However, there is an increase in probability for forces less than half of the mean contact force. Meanwhile, in the wide gradated assemblies, contact forces have an exponential decrease in the probability distribution, and the probability of weak contact forces is higher than that in the narrow PSD packings. For a wide PSD, finer particles may fill the voids between larger grains. Stronger forces may only be supported by large particles, and finer grains mainly transmit relatively weak forces. This may lead weak contact forces to have a larger quantity than the strong forces.

Liu et al. [3] carried out an experimental test on mono-sized glass beads in a cylinder to measure strong force chains in the granular material. The glass beads are in an isotropic stress condition. The force distribution is measured by carbon paper on the inside surface of the container, and their results showed that the probability density distribution of the normalized forces decreased exponentially with an increase in the normalized force. They also assumed a simple theoretical model whereby the inhomogeneity of the particle arrangement causes unequal distributions of force transmission, resulting in strong force chains (see also [40]). By stochastic analysis, they proposed that the probability density distribution of contact forces for the isotropic glass beads at the static state can be written as:

(16) $$P\left(f\right)=\frac{C^{C}}{(C-1)!}f^{C-1}\;exp\;\left(-Cf\right)$$

where *f* is the normalized contact force and *C* is a constant related to the particle arrangement. From our simulation results without considering gravity, in the conventional triaxial path, when the dry granular materials reach the critical state, the only controlling parameter of the contact force distribution should be the particle gradation. Empirically, we proposed a probability function of the contact forces at the critical state in a similar exponential form:

(17) $$P\left(f\right)=K^{K}f^{K-1}exp\left(-Kf\right)$$

where *K* is a parameter related to the particle size distribution. For a well-graded packing, it is determined as:

(18) $$K=\frac{3}{2}{\left(\frac{2}{3}\right)}^{\frac{d_{max}-d_{min}}{\bar{d}}}$$

in which $d_{max}$ and $d_{min}$ are the maximum and minimum particle diameters in the assembly respectively and $\bar{d}$ is the mean particle size.

Equation (17) predicted contact force distributions are also plotted in Figure 8 to compare with the numerical experiment results. It can be seen that, for both the narrow and wide gradations, Equation (17) predicted curves showed good agreement. Mathematically, if the gradation becomes even wider, the *K* value will be reduced, but Equation (17) will produce a higher probability for weak contact forces. This is consistent with the expectations that for wider PSDs there will be more particles filling in the voids among the larger grains.

## 4. Force Transmission of Wet Granular Materials

### 4.1. Capillary Effect on Mechanical Behaviors

Wet granular materials are also simulated in triaxial test. As can be seen in the previous section that by using the Hooke model the ultimate void ratio may be slightly higher, the wet granular material is thus simulated by the Hertz contact model with the capillary bridge effect coupled. The particle size distribution of the material is the narrow distribution in Figure 3. Two dry specimens with different void ratios (e=0.63 and e=0.73) are firstly prepared. The specimens are isotropically compressed to 10 kPa. Then, the capillary bridge model is applied with the boundary stress maintained. The matric suction value is 20 kPa throughout the sample, which leads the degree of saturation in the sample to be around 10%. It is observed that the void ratio is not obviously altered by switching on the water bridge effect.

**Figure 9:**
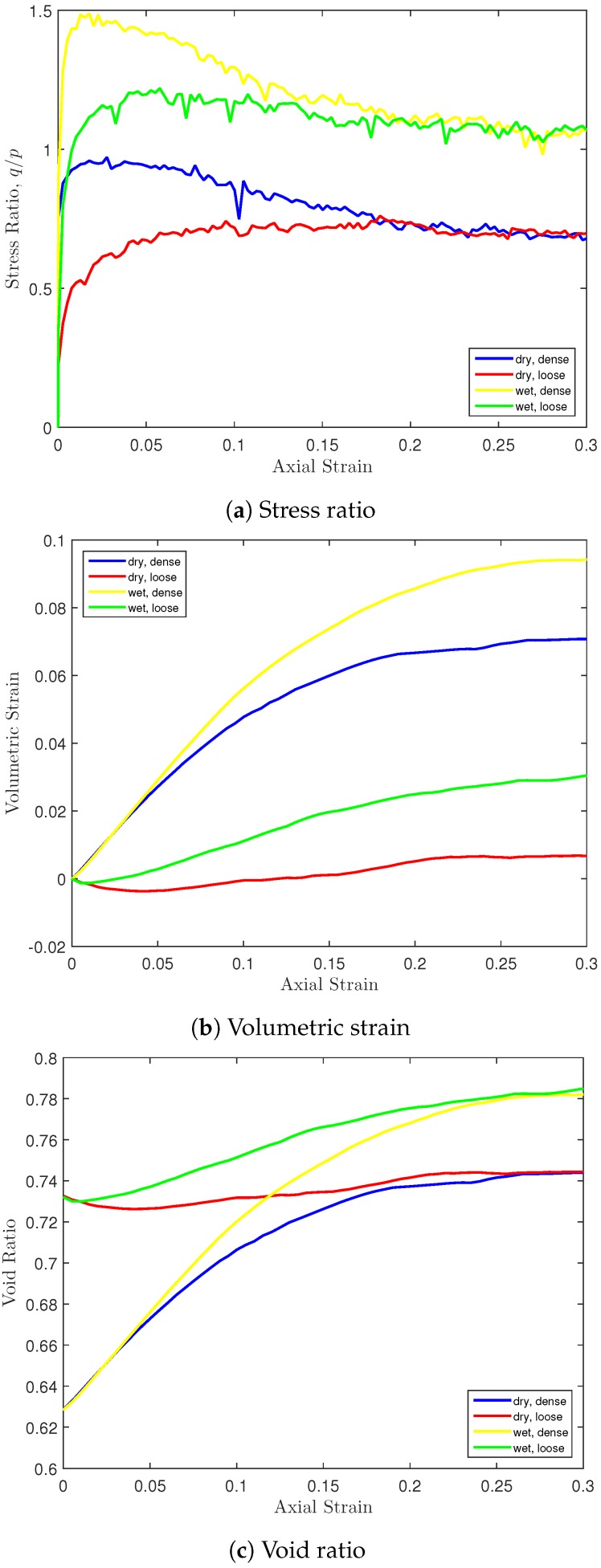
Stress-strain behaviors of dry and wet samples.

The mechanical behaviors (stress ratio, volumetric strain and void ratio) of the dry and wet granular materials in the triaxial test under 10 kPa confinement are presented in Figure 9. It can be seen that the capillary effect significantly increased the material strength. Independent of the initial void ratio, the determined ultimate (critical state) stress ratios exist for dry and wet granular materials, respectively. The capillary effect increased the critical state stress ratio by 0.5. The cohesive capillary force also increased the material dilatancy significantly. It can be seen that the ultimate volumes of wet materials are much higher. Correspondingly, void ratios for wet granular materials are also higher in triaxial shearing. For dense and loose granular materials, the void ratio values converge to the same at critical state. However, the critical state void ratio of wet granular materials is larger due to the higher dilatancy.

### 4.2. Force Transmission Pattern with Cohesive Capillary Effect

According to the discussion in the previous section, dry granular materials reaching the critical state have a unique distribution of inter-particle interactions including normal and tangential inter-particle forces, meaning the force transmission pattern at the critical state is unique. A similar investigation can also be carried out for the wet granular materials. The micro statistic investigation was implemented on both the loose and dense samples. The corresponding non-cohesive dry material results were also analyzed as references for the wet material behaviors.

**Figure 10:**
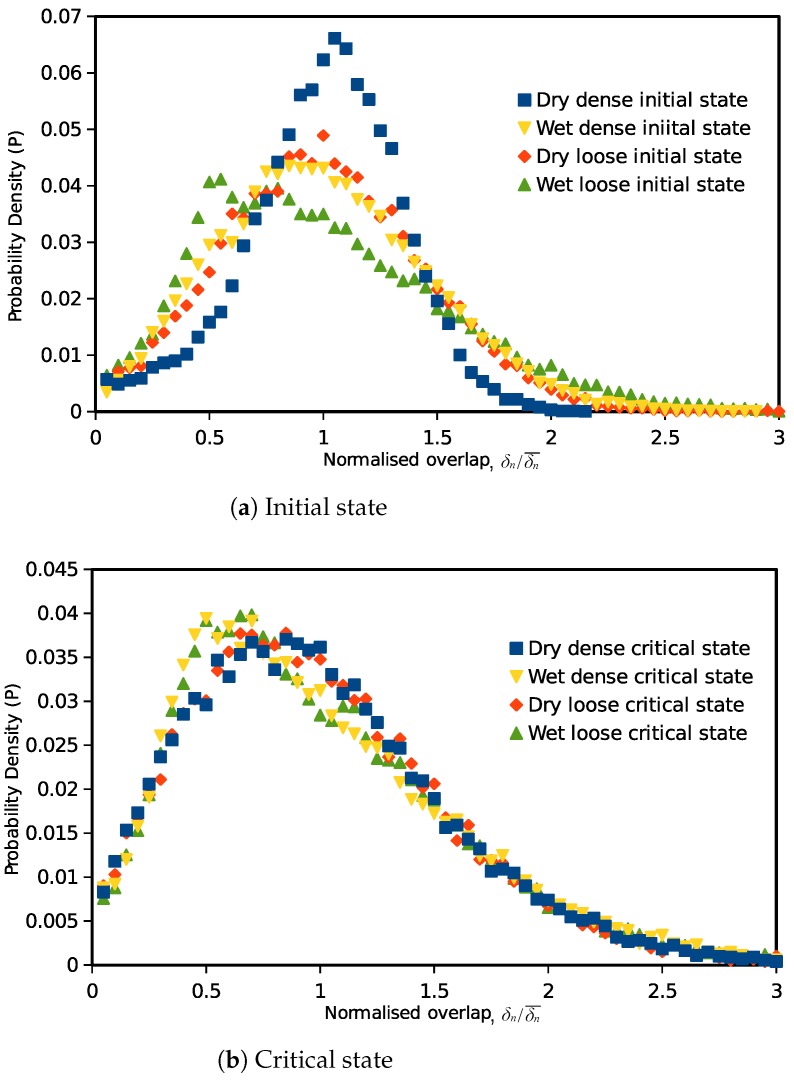
Initial and critical state probability distribution of normalized overlaps ($\delta_{n}/\bar{\delta_{n}}$) in dry and wet conditions.

Figure 10 represents the initial and critical state probability density distributions of the normalized inter-particle overlaps for the dry and wet granular materials. The probability is calculated on the basis of 60 equal-sized intervals between zero and three. At the isotropic (initial) state, overlaps in dry granular materials showed a typical normal distribution, and the distribution is wider in the loose specimen. By adding the cohesive capillary effect, the inter-particle overlap distribution in the dense packing spreads wider than that in the dry specimen, which indicates that a greater amount of smaller or larger overlapping occurred, although the total sample volume is not altered obviously. Meanwhile, in the loose sample, the capillary effect moves the distribution peak to a smaller value. It is also noticed that in wet materials, the distributions are no longer in normal distributions. After the specimens are sheared to the critical state, unique distributions for normalized overlaps exist in both the dry and wet granular materials. The distribution in dry material is similar to a normal distribution. However, in the wet materials, the distribution peak is moved from one to 0.5. The probability is a linear increase trend in small overlaps (less than 0.5), followed by the exponential decay in medium and large overlaps (in the range bigger than 0.5).

**Figure 11:**
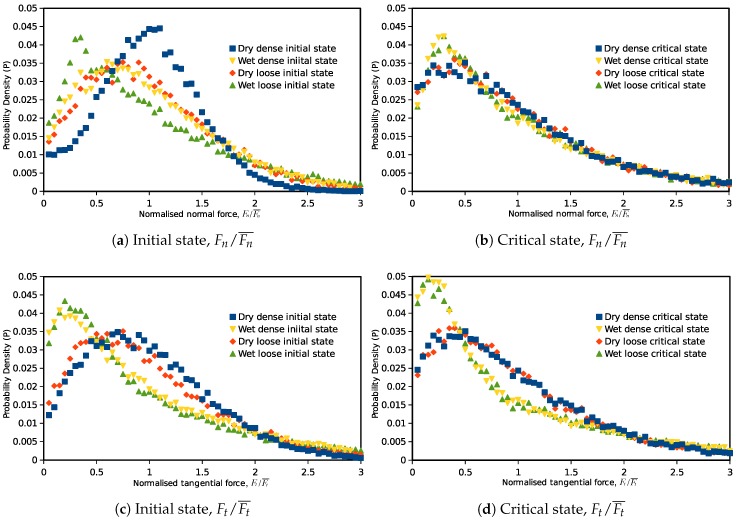
Initial and critical state probability distribution of inter-particle forces in dry and wet granular materials.

Probability density distributions of inter-particle forces (normalized normal and tangential forces) in dry and wet granular materials at the initial and critical states are plotted in Figure 11. The normal force here is the normal component of the total inter-particle force so that the attractive capillary forces are also included. At the initial state, corresponding to the inter-particle overlap distribution, the distribution of normal contact forces in the dry granular material also seems to be a normal distribution. For the wet samples at the initial state, the normal force value with the highest possibility in the assembly is less than the mean value, and there are more weak normal forces (normalized value less than one) in the assembly. This indicates that the capillary forces increased the total quantity of the inter-particle contacts (increased the coordination number), and there are more weak forces on the newly-formed contacts. For tangential forces, the probability distribution is less relevant to the sample void ratio. In the wet granular material, the weak tangential forces also have a higher probability. For both the normal and tangential forces, relatively weaker and stronger forces (normalized value <0.5 and >2) have a higher probability in wet samples than those in dry specimens.

After the specimens are sheared to the critical state, both the normal and tangential forces showed a unique critical state distribution independent of their initial void ratios. Unlike the unique force distribution in dry granular materials, in cohesive materials, the probability distributions of normal and tangential inter-particle forces no longer have the same pattern at the critical state. In a wet sample, the normal contact force distribution is closer to that of a dry material, with a slight difference in weak forces (less than half the mean force). However, the tangential force distribution in wet soils is quite different from the distribution in a dry sample. There are a large number of small shear forces existing on the contacts at the critical state. These indicate that the macro mechanical behavior change induced by the cohesive capillary effect is more correlative to the microscopic tangential inter-particle force distribution patterns.

### 4.3. Capillary Effect on Stress Transmission

The stress transmission patterns in the dry and wet granular materials are investigated by analyzing the stress on each particle. By following the stress tensor definition by [41] and limiting the stress volume as the particle volume, the stress tensor for a particle in contact with particles from the set *P* can be written as:

(19) $$\sigma_{ij}^{P}=\frac{3}{4\pi R^{3}}\sum_{a\in P}n_{i}^{a}f_{j}^{a}$$

where *R* is the particle radius, $n_{i}^{a}$ is the unit vector pointing from the *a*-th force acting point on the particle to the particle center and $f_{i}^{a}$ is the *a*-th force. The mean stress on a particle can then be evaluated by $\frac{1}{3}tr\left(\sigma_{ij}^{P}\right)$. A positive mean stress means the particle is in compression, and a negative mean stress means tensile stress on the particle. For dry materials, particles are all in compression. However, when the cohesive capillary effect is applied, some particles may have a tensile effect.

**Figure 12:**
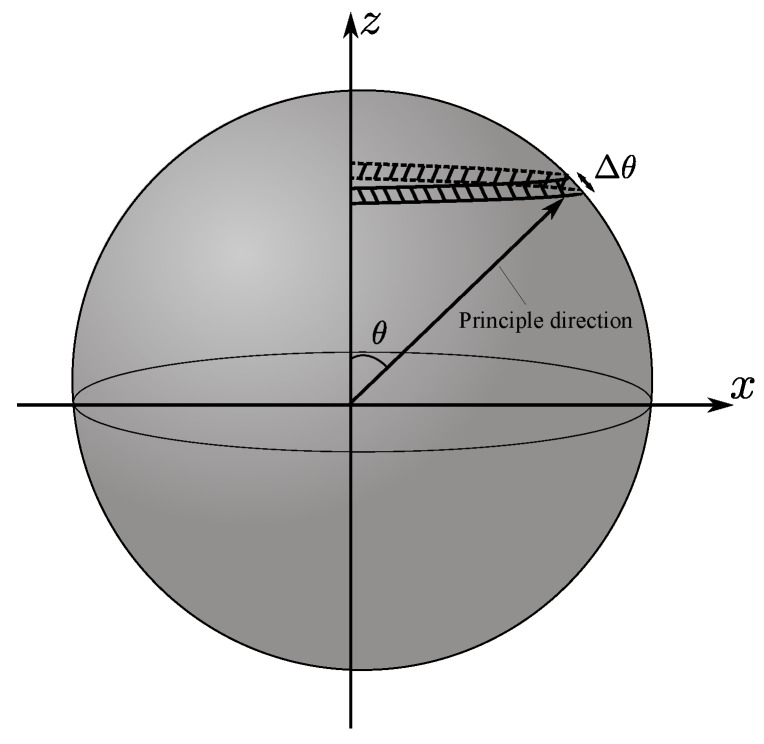
Principal directions on the unit sphere.

By transforming the particle stress tensors to principal stresses, the major principal stress directions of particles can be investigated to understand the stress transmission patterns in dry and wet granular materials. As the stress on the specimen is axisymmetric, the three-dimensional distribution of the principal directions can be converted to a two-dimensional distribution. For each particle, there are two major principal directions, which are opposite of each other. All of the major principal directions can be graphically expressed on a unit sphere in Figure 12. The *z* axis is the direction in which the axial strain is applied. The probability of a particular major principal direction with an angle θ to the *z* axis is estimated based on the half circle band on the unit sphere with a tiny width of Δθ. Thus, the probability of a particular direction is estimated as:

(20) $$P\left(\theta \right)=\frac{N_{\theta }}{\pi sin\theta N\Delta \theta }$$

where $N_{\theta }$ is the number of principal directions within the half circle band and *N* is the total number of principal directions (two times the total grain number). With this estimation method, the directional distribution of three-dimensional data is converted to the *x*-*z* plane.

**Figure 13:**
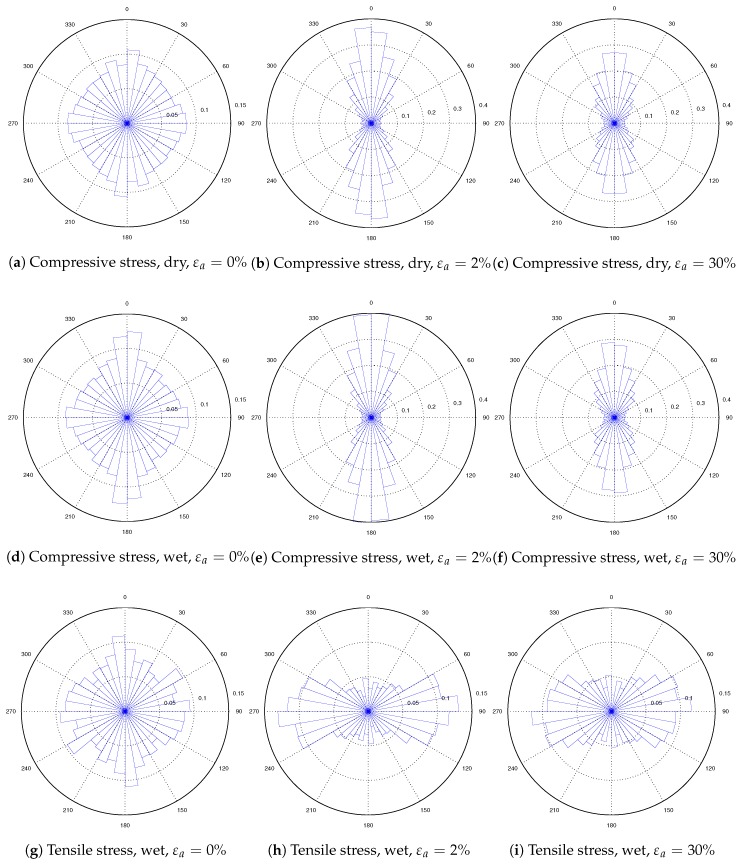
Directional distribution of compressive and tensile stress principal directions.

The directional distribution of the major principal directions on particles in the dense specimen is analyzed to demonstrate how the compressive and tensile stresses are transmitted in the dry and wet granular materials in triaxial shearing (Figure 13). The initial state ($\varepsilon_{a}=0\%$), the peak state ($\varepsilon_{a}=2\%$) and the critical state ($\varepsilon_{a}=30\%$) are investigated. In the dry material, at the initial state, the principal directions of stresses on particles are almost isotropically distributed in all directions. The probability is around $\frac{1}{4\pi }$ for all directions. With the axial strain increase, the principal directions of compressive stresses become anisotropic, and they are mainly distributed along the axial direction. The anisotropy of the principal directions is larger at the peak state than that at the critical state. In the wet specimen ($S_{r}=1.7\%$), the directional distribution of the principal directions of the compressive stresses is similar to that in the dry specimen, but the anisotropy of principal directions at peak strength is higher than that in the dry one. Furthermore, tensile stresses on particles at the initial state are also isotropically distributed. However, upon triaxial shearing, the tensile stresses on particles are mainly acting through the horizontal direction. These phenomena indicate that the capillary bridge-induced tensile stress plays a significant role in mechanical behaviors. The capillary effect adds an additional tensile resistance on the horizontal plane, which increased the material strength.

## 5. Conclusions

This paper explored the force transmission modes of non-cohesive dry and cohesive wet granular materials. The contact model effect was focused on. Both linear and non-linear models are applied in the dry material simulation, and a capillary bridge mode incorporated with the Hertz model is used to simulate the wet granular material. Samples with different initial void ratios were sheared by following the conventional triaxial loading path, and the initial and critical state inter-particle force and stress distributions were analyzed. Conclusions can be summarized from the dry and wet material results.

For non-cohesive dry granular materials:

1. Using either the Hooke or Hertz contact model does not alter the stress-strain behaviors of a specimen with a nearly mono-sized particle size distribution if it is sheared from the same dimensionless stress state. However, if the grain gradation becomes wider, discrepancies between these two models increase as the critical state void ratio of the Hooke model result is higher.
2. At the initial state (isotropic state), both the inter-particle overlaps and inter-particle forces are in normal distributions. The void ratio and particle gradation may affect the width of the distribution. Additionally, when the material reaches the critical state, the probability of relatively weak forces (forces less than average) is increased.
3. There is a unique critical state force transmission pattern for a non-cohesive granular material independent of the contact model. The normal and tangential force distributions can be formulated by a certain probability density function, which is only related to the grain size distribution.

Additionally, for cohesive wet materials:

1. The material strength and dilatancy are higher than those of the non-cohesive material. The critical state also exists for a wet granular material, and the critical state stress ratio and void ratio are increased by the inter-particle attractive force.
2. The cohesive force between particles alters the contact force distribution. There are more weak forces (forces less than the average force) in the cohesive materials at both the initial and ultimate states.
3. Corresponding to the critical state strength and void ratio, the dense and loose materials have the same contact force distribution at the critical state. However, the distribution is different from those of non-cohesive samples, as the probability of weak forces is higher.
4. Particles in non-cohesive materials are mainly under compressive stresses. However, in cohesive materials, some grains are in tensile stresses. Upon triaxial loading, the principal directions of compressive stresses are mainly in the axial direction, and the tensile stresses are acting in the horizontal plane. The additional confinement by the tensile stress increased the material strength and dilatancy.

## Figures and Tables

**Table 1:** Summary of the parameters used for the DEM simulations. RVE, representative volume element.

RVE Length	$\bar{d}$ (mm)	μ	ρ (kg/m${}^{3}$)	Contact Model	Other Parameters		
$20\;\bar{d}$	0.02	0.5	2500	Hertz (dry and wet)	*E* (GPa)	ν	$e_{r}$
					70	0.25	0.2
				Hooke (dry)	$k_{n}$ (N/m)	$k_{t}$ (N/m)	$\gamma_{n}$ and $\gamma_{t}$
					$10^{5}$	$10^{5}$	0.7

## References

[1] Drescher A., de Josselin de Jong G. (1972). Photoelastic verification of a mechanical model for the flow of a granular material. J. Mech. Phys. Solids.

[2] Mueth D., Jaeger H., Nagel S. (1998). Force distribution in a granular medium. Phys. Rev. E.

[3] Liu C.H., Nagel S.R., Schecter D.A., Coppersmith S.N., Majumdar S., Narayan O., Witten T.A. (1995). Force Fluctuations in Bead Packs. Science.

[4] Ostojic S., Somfai E., Nienhuis B. (2006). Scale invariance and universality of force networks in static granular matter. Nature.

[5] Cundall P.A., Strack O.D.L. (1979). A discrete numerical model for granular assemblies. Géotechnique.

[6] Radjai F., Jean M., Moreau J., Roux S. (1996). Force Distributions in Dense Two-Dimensional Granular Systems. Phys. Rev. Lett..

[7] Thornton C., Antony S. (1998). Quasi-static deformation of particulate media. Philos. Trans. R. Soc. A Math. Phys. Eng. Sci..

[8] Antony S. (2000). Evolution of force distribution in three-dimensional granular media. Phys. Rev. E.

[9] Antony S.J. (2007). Link between single-particle properties and macroscopic properties in particulate assemblies: Role of structures within structures. Philos. Trans. Ser. A Math. Phys. Eng. Sci..

[10] Schofield A., Wroth P. (1968). Critical State Soil Mechanics.

[11] Luding S. (2008). Cohesive, frictional powders: Contact models for tension. Granul. Matter.

[12] Richefeu V., El Youssoufi M., Radjaï F. (2006). Shear strength properties of wet granular materials. Phys. Rev. E.

[13] Soulié F., El Youssoufi M.S., Cherblanc F., Saix C. (2006). Capillary cohesion and mechanical strength of polydisperse granular materials. Eur. Phys. J. E Soft Matter.

[14] Richefeu V., El Youssoufi M.S., Peyroux R., Radjaï F. (2008). A model of capillary cohesion for numerical simulations of 3D polydisperse granular media. Int. J. Numer. Anal. Methods Geomech..

[15] Scholtès L., Hicher P., Nicot F., Chareyre B., Darve F. (2009). On the capillary stress tensor in wet granular materials. Int. J. Numer. Anal. Methods Geomech..

[16] Gabrieli F., Lambert P., Cola S., Calvetti F. (2012). Micromechanical modeling of erosion due to evaporation in a partially wet granular slope. Int. J. Numer. Anal. Methods Geomech..

[17] Richefeu V., Radjaı F., El Youssoufi M.S. (2006). Stress transmission in wet granular materials. Eur. Phys. J. E.

[18] Richefeu V., El Youssoufi M.S., Azéma E., Radjaï F. (2009). Force transmission in dry and wet granular media. Powder Technol..

[19] Wheeler S.J., Sivakumar V. (1995). An elasto-plastic critical state framework for unsaturated soil. Géotechnique.

[20] Toll D.G., Ong B.H. (2003). Critical-state parameters for an unsaturated residual sandy clay. Géotechnique.

[21] Toll D.G., Ali Rahman Z. (2017). Critical state shear strength of an unsaturated artificially cemented sand. Géotechnique.

[22] Kloss C., Goniva C., Hager A. (2012). Models, algorithms and validation for opensource DEM and CFD-DEM. Progress Comput. Fluid Dyn..

[23] Hertz H. (1882). Über die berührung fester elastischer körper. J. Reine Angew. Math..

[24] Johnson K.L. (1987). Contact Mechanics.

[25] Brilliantov N., Spahn F., Hertzsch J.M., Pöschel T. (1996). Model for collisions in granular gases. Phys. Rev. E.

[26] Silbert L., Ertaş D., Grest G., Halsey T., Levine D., Plimpton S. (2001). Granular flow down an inclined plane: Bagnold scaling and rheology. Phys. Rev. E.

[27] Zhang H.P., Makse H.A. (2005). Jamming transition in emulsions and granular materials. Phys. Rev. E.

[28] Wang J.P. (2015). Discrete Element Modeling and Micromechanics of Pendular State Unsaturated Granular Materials. Ph.D. Thesis.

[29] Wang J.P., Li X., Yu H.S. (2017). A micro-macro investigation of the capillary strengthening effect in wet granular materials. Acta Geotech..

[30] Mindlin R. (1949). Compliance of Elastic bodies in contact. J. Appl. Mech..

[31] Wang J.P., Li X., Yu H.S. (2017). Stress-Force-Fabric Relationship for Unsaturated Granular Materials in Pendular States. J. Eng. Mech..

[32] Wang J.P., Gallo E., François B., Gabrieli F., Lambert P. (2017). Capillary force and rupture of funicular liquid bridges between three spherical bodies. Powder Technol..

[33] Hotta K., Takeda K., Iinoya K. (1974). The capillary binding force of a liquid bridge. Powder Technol..

[34] Rothenburg L., Bathurst R.J. (1992). Micromechanical features of granular assemblies with planar elliptical particles. Géotechnique.

[35] Jiang M., Konrad J., Leroueil S. (2003). An efficient technique for generating homogeneous specimens for DEM studies. Comput. Geotech..

[36] Cates M., Wittmer J., Bouchaud J.P., Claudin P. (1998). Jamming, Force Chains, and Fragile Matter. Phys. Rev. Lett..

[37] Agnolin I., Roux J.N. (2007). Internal states of model isotropic granular packings. I. Assembling process, geometry, and contact networks. Phys. Rev. E.

[38] Padbidri J.M., Mesarovic S.D. (2011). Acceleration of DEM algorithm for quasistatic processes. Int. J. Numer. Methods Eng..

[39] Yousefi A., Ng T.T. (2017). Dimensionless input parameters in discrete element modeling and assessment of scaling techniques. Comput. Geotech..

[40] Coppersmith S.N., Liu C.H., Majumdar S., Narayan O., Witten T.A. (1996). Model for force fluctuations in bead packs. Phys. Rev. E.

[41] Christoffersen J., Mehrabadi M.M., Nemat-Nasser S. (1981). A Micromechanical Description of Granular Material Behavior. J. Appl. Mech..

